# Associations of Brain-Derived Neurotropic Factor rs6265 Gene Polymorphism with Personality Dimensions among Athletes

**DOI:** 10.3390/ijerph19159732

**Published:** 2022-08-07

**Authors:** Kinga Humińska-Lisowska, Jolanta Chmielowiec, Krzysztof Chmielowiec, Marta Niewczas, Milena Lachowicz, Paweł Cięszczyk, Jolanta Masiak, Aleksandra Strońska-Pluta, Monika Michałowska-Sawczyn, Ewelina Maculewicz, Anna Grzywacz

**Affiliations:** 1 Faculty of Physical Culture, Gdansk University of Physical Education and Sport, 80-336 Gdansk, Poland; 2 Department of Hygiene and Epidemiology, Collegium Medicum, University of Zielona Góra, 28 Zyty St., 65-046 Zielona Góra, Poland; 3 Faculty of Physical Education, University of Rzeszów, 35-959 Rzeszów, Poland; 4 Department of Psychology, Gdansk University of Physical Education and Sport, 80-336 Gdansk, Poland; 5 Second Department of Psychiatry and Psychiatric Rehabilitation, Medical University of Lublin, 1 Głuska St., 20-059 Lublin, Poland; 6 Independent Laboratory of Health Promotion, Pomeranian Medical University in Szczecin, 11 Chlapowskiego St., 70-204 Szczecin, Poland; 7 Faculty of Physical Education, Jozef Pilsudski University of Physical Education in Warsaw, 00-809 Warsaw, Poland

**Keywords:** genes, athletes, personality, *BDNF* gene, rs6265

## Abstract

Brain-Derived Neurotropic Factor (*BDNF*) is one of the essential mediating factors of exercise-induced neuroplasticity, but the underlying molecular mechanisms of exercise-induced neuroplasticity are still largely unknown. Personality dimensions differentiate individuals and depend on genes and environmental factors. The dimensions of openness to experience, emotional stability, extraversion and conscientiousness have been reported to be positively related to performance; considering agreeableness, a negative relation with sports performance was emphasized. However, not enough effort has been put into investigating the relationship between genetic polymorphisms affecting psychological abilities and competitive power sports. The aim of this study was to investigate the association of the rs6265 polymorphism of *BDNF* with personality dimensions in martial arts athletes. The study was conducted among martial arts athletes. The study group included 258 volunteers (martial arts athletes (*n* = 106) and controls (*n* = 152). *BDNF* polymorphism testing was performed using the real-time PCR method; personality dimensions were assessed using standardized NEO-FFI questionnaires. All analyses were performed using STATISTICA 13. We observed that martial arts athletes’ G/G genotypes compared to the control group G/G genotypes presented significantly higher severity of personality dimension “conscientiousness”. In comparison with the controls, the case group subjects had significantly higher scores in the dimension extraversion (M 6.89 vs. M 6.43, *p* = 0.0405) and conscientiousness/scale (M 7.23 vs. M 5.89, *p* < 0.0001). The results of 2 × 3 factorial ANOVA noticed a statistically significant effect of combined factor *BDNF* rs6265 genotype of martial arts/control (F_2,252_ = 3.11, *p* = 0.0465, η^2^ = 0.024). Additionally, we observed that the results of 2 × 3 factorial ANOVA showed a statistically significant influence of combined factor *BDNF* rs6265 of genotype martial arts/ control (F_2,252_ = 6.16, *p* = 0.0024, η^2^ = 0.047). The combination of the analysis of personality dimensions with genetics—as in the case of the polymorphism of the *BDNF* gene related to neuroplasticity—indicates that neurobiology cannot be ignored in educating sports champions. We already know that this is related to genetics. However, little is still known about the influence of personality traits on sports performance. We observed that martial arts athletes’ G/G genotypes, in comparison to the control group’s G/G genotypes, presented significantly higher severity of personality dimension “conscientiousness”. This is worthy of further analysis and probably longitudinal studies on a more numerous group of athletes.

## 1. Introduction

“Athletic phenotype” is complex and depends on a combination of many elements, such as environmental variables and experience (training and diet) and biological and genetic factors. In recent decades, “sports genomics” has played an important role, showing that some DNA Single Nucleotide Polymorphisms (SNPs) can be linked to athlete performance and championship level, having an impact on physical activity behavior, endurance, speed, strength, flexibility, power, neuromuscular coordination, energetic expenditure, metabolic and cardiorespiratory fitness, as well as personality traits [1,2].

Personality is defined as consistent patterns of thoughts, emotions, and behaviors that characterize a person across a lifetime [3]. The NEO Five-Factor Personality Inventory (NEO FFI) is a questionnaire that measures a comprehensive model of general personality traits. The Five-Factor Model, or “Big Five”, defines the five dimensions of personality: extraversion, neuroticism, openness to experience, conscientiousness, and agreeableness. They depend on genes and environmental factors. They are stable over time and across cultures [4]. Personality traits appeared to be associated with the psychological characteristics of athletes [5,6]. Conscientiousness, openness, and agreeableness were found to be significant predictors of sports performance [7].

Previous studies looked at personality traits in various sports disciplines and found differences among different types of sports, race, gender, experience, and skill level [8,9,10]. Furthermore, regarding the personality traits of martial arts practitioners, it was found that openness to experience, extraversion, emotional stability, and conscientiousness are positively related to performance. At the same time, agreeableness displays a negative relation with sports performance [11].

Numerous studies indicate that physical exercise improves brain function and structure [12,13,14,15,16]. In the last decades, Brain-Derived Neurotropic Factor (BDNF) has been extensively studied in neuroscience as one of the essential mediators of exercise-induced neuroplasticity. However, the underlying molecular mechanisms of exercise-induced neuroplasticity are still barely known [17]. Proposed mechanisms of exercise-induced neuroplasticity are increased expression, secretion, and downstream signaling of neurotrophic factors (among them BDNF), reduced stress levels [18], reduced inflammation [19,20], and improved metabolic and cardiovascular parameters. BDNF promotes neuronal survival and differentiation, as well as regulates synaptic transmission and plasticity (synaptogenesis, neurogenesis, long-term potentiation) of the adult brain in many regions of the Central Nervous System (CNS) [21]. First, a positive correlation between physical activity and *BDNF* expression was observed in rodents [22]. Pharmacological blocking of BDNF signaling in the rodent hippocampus reduced the neuroplastic effects of exercise. Similar to rodents, physical activity (chronic and acute) increases peripheral BDNF levels in healthy humans [23,24,25,26].

Our research has concentrated on the case-control analysis of athletes’ groups in the aspect of their personality traits in association with rs6265 *BDNF* gene polymorphism. This common G196A nonsynonymous polymorphism rs6265 that produces a valine (Val) to methionine (Met) substitution at codon 66 (Val66Met), is present in about 20% of the total world population, with strong differences among different ethnicities (e.g., 22% in Caucasians, 2% in African Americans) (IGSR: The International Genome Sample Resource, 2021). Rs6265 polymorphism is shown to modulate BDNF secretion and its distribution within a cell, synaptic plasticity, and negative influence on memory, cognitive function, and vulnerability to psychological stress [27,28,29]. This is probably because the discharge induced by depolarizing stimuli is lower in *BDNF* AA carriers than in *BDNF* GG carriers [30]. Additionally, it was already observed that *BDNF* rs6265 polymorphism increases the risk for depression. However, this genetic risk factor for suicidal ideation and major depression may be reduced by physical activity [31,32]. This is why there is an assumption that athletes with superior athletic performance are less likely to be *BDNF* AA carriers, which is also associated with an increased vulnerability to psychological stress. BDNF seems to be one of the most crucial neurotrophins engaged in sports performance. Several research groups have already confirmed such observations [29,33,34,35,36,37,38,39,40]. 

In our study, we investigate the association of the rs6265 polymorphism of *BDNF* with personality dimensions in martial arts athletes. We decided to explore martial arts genotypes compared to the control group and the severity of personality dimensions.

## 2. Materials and Methods

### 2.1. Subjects

The study group consisted of 258 volunteers: martial arts (*n* = 106; mean age = 23.33 SD = 5.76, Minimum 17.00, Maximum 35.00, men 79%, women 21%, judo, *n* = 25; boxing, *n* = 10; kickboxing, *n* = 14; Ju-jitsu, *n* = 35; wrestling, *n* = 22) and healthy, non-athlete controls (*n* = 152; mean age = 22.23, SD = 4.55, Minimum 17.00, Maximum 50.00, men 85%, women 15%). A total of 38% of the martial arts group achieved the championship level. The research was conducted in 2018–2019, before the COVID-19 pandemic. The study was conducted in accordance with the Declaration of Helsinki principles and approved by the by Bioethics Committee for Clinical Research of the Regional Medical Society in Szczecin (protocol nr 13/KB/VI/2016, 8 December 2016). All subjects provided signed informed consent for participating in the research. There was no financial or other compensation for being part of the study sample. Martial arts and control subjects were examined by the NEO Five-Factor Personality Inventory (NEO-FFI).

The NEO-FFI includes six dimensions for each of the five traits—extraversion (positive emotion, warmth, gregariousness, activity, excitement seeking, assertiveness), agreeableness (tendermindedness, trust, altruism, straightforwardness, compliance, modesty), openness to experience (fantasy, feelings, aesthetics, actions, values, ideas), conscientiousness (deliberation, competence, dutifulness, order, achievement striving, self-discipline), neuroticism (anxiety, vulnerability to stress, hostility, self-consciousness, impulsiveness, depression) [41].

The results of NEO-FFI inventories were given as sten scores. The conversion of the raw score into the sten scale was performed according to Polish norms for adults; it was assumed that: stens: 1–2—very low scores; 3–4—low scores, 5–6—average scores; 7–8—high scores, 9–10—very high scores. 

### 2.2. Genotyping

Using standard procedures, the genomic DNA was extracted using the High Pure PCR Template Preparation Kit (Roche, Basel, Switzerland) from venous blood. Rs6265 polymorphism was conducted using TaqMan SNP Genotyping Assay C__11592758_10 (ThermoFisher Scientific, Waltham, MA, USA) with the real-time PCR method.

A LightCycler^®^ 480 II System (Roche Diagnostic, Basel, Switzerland) was applied to perform the fluorescence resonance energy in the genotypic data. The data relating to the *BDNF* gene polymorphism were obtained under the following conditions: PCR was performed with 50 ng DNA of each sample in a final volume of 20 µL containing 2 µL reaction mix, 0.5 mM of each primer, 0.2 mM of each hybridization probe, and 2 mM MgCl2, according to the manufacturer’s instructions, with initial denaturation (95 °C for 10 min) and then 35 cycles of denaturation (95 °C for 10 s), annealing (60 °C for 10 s), and extension (72 °C for 15 s). After amplification, a melting curve was generated by holding the reaction at 40 °C for 20 s and then heating slowly to 95 °C. The fluorescence signal was plotted against temperature to provide melting curves for each sample.

### 2.3. Statistical Analysis

Concordance between the genotype frequency distribution and Hardy–Weinberg equilibrium (HWE) was tested with the HWE software (https://wpcalc.com/en/equilibriumhardy-weinberg/ (accessed on 10 May 2022)). The relationships between *BDNF* rs6265, martial arts and control subjects and NEO Five-Factor Inventory were analyzed in a multivariate analysis of Factor effects ANOVA (NEO-FFI/ × genetic feature × control and martial arts × (genetic feature × control and martial arts)). The homogeneity of variance was satisfied (Levene test *p* > 0.05). The distribution of the analyzed variables did not present a normal distribution. The NEO-FFI (neuroticism, extraversion, openness, agreeability, and conscientiousness) was measured and compared using the Mann–Whitney U test. *BDNF* rs6265 genotype frequencies between healthy control subjects and martial arts subjects were tested with the chi-square test. For these variables, the Bonferroni multiple comparisons correction was applied, and the accepted level of significance was 0.01 (0.05/5) and 0.0083 (0.05/6). All computations were performed with the usage of STATISTICA 13 (Tibco Software Inc, Palo Alto, CA, USA) for Windows (Microsoft Corporation, Redmond, WA, USA).

## 3. Results

The frequency distributions accorded with the HWE. There was no statistical difference between martial arts participants and people from the control group (Table 1).

The *BDNF* rs6265 genotypes and alleles frequencies in the studied sample do not differ in the analyzed groups of subjects (Table 1).

The means and standard deviations for NEO Five-Factor Inventory results in groups of martial arts subjects and control subjects are presented in Table 2. In comparison with the controls, the case group subjects had significantly higher scores on the extraversion/scale (M 6.89 vs. M 6.43, *p* = 0.0405) and conscientiousness/scale (M 7.23 vs. M 5.89, *p* < 0.0001).

Neuroticism/scale and BDNF rs6265.

**Figure 1:**
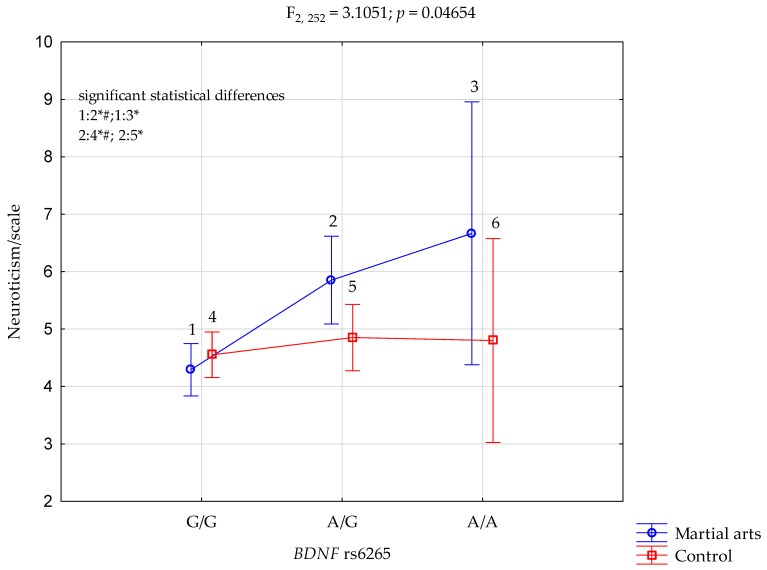
Interaction between martial arts/-Control and *BDNF* rs6265 and Neuroticism scale. # Bonferroni correction was used, and the *p*-value was reduced to 0.0083 (*p* = 0.05/6 (number of statistical tests conducted)). G/G and A/A—genotypes (homozygotes), A/G—genotype (heterozygote), G and A—alleles, 1—G/G, 2—A/G, 3—A/A, 4—G/G, 5—A/G, 6—A/A, *BDNF*—Brain-Derived Neurotropic Factor, F—F ratio score, * significant result *p* = 0.05, *p*—*p*-value.

The results of 2 × 3 factorial ANOVA noticed statistically significant effect (without Bonferroni correction) of combined factor *BDNF* rs6265 genotype of martial arts/control (F_2,252_ = 3.11, *p* = 0.0465, η^2^ = 0.024) (Table 3, Figure 1). Power calculation—our sample had more than 59% power to detect the combined factor of martial arts/control × *BDNF* rs6265 and their interaction effect (about 2.4% of the phenotype variance).

Conscientiousness/scale and BDNF rs6265. 

**Figure 2:**
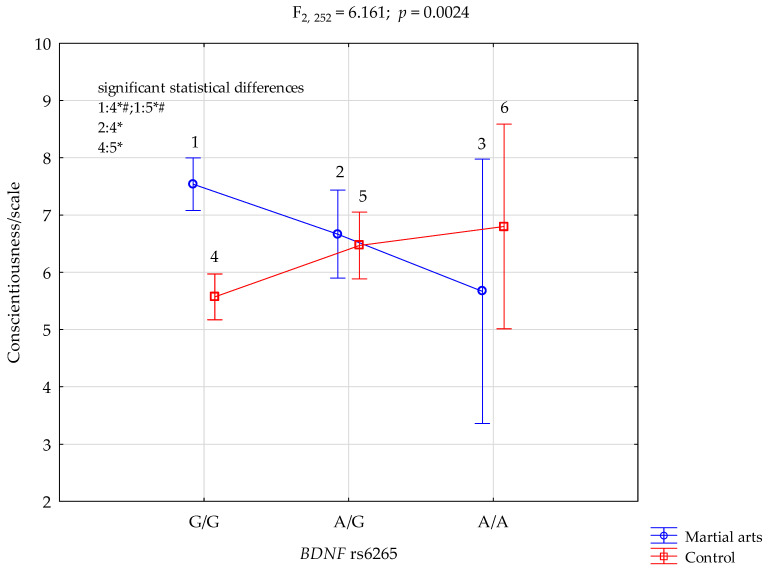
Interaction between martial arts/control and *BDNF* rs6265 and conscientiousness scale. # Bonferroni correction was used, and the *p*-value was reduced to 0.0083 (*p* = 0.05/6 (number of statistical tests conducted)). G/G and A/A—genotypes (homozygotes), A/G—genotype (heterozygote), G and A—alleles, 1—G/G, 2—A/G, 3—A/A, 4—G/G, 5—A/G, 6—A/A., *BDNF*—Brain-Derived Neurotropic Factor, F—F ratio score, * significant result *p* = 0.05, *p*—*p*-value.

The results of 2 × 3 factorial ANOVA noticed a statistically significant effect (with Bonferroni correction) of combined factor *BDNF* rs6265 genotype of martial arts/control (F_2,252_= 6.16, *p* = 0.0024, η^2^ = 0.047) (Table 3, Figure 2). Our sample had more than 89% power to detect the combined factor of martial arts/control x *BDNF* rs6265 and their interaction effect (about 4.7% of the phenotype variance). The post-hoc analysis is shown in Table 4.

## 4. Discussion

In our study, we investigate the association of the rs6265 polymorphism of *BDNF* with personality dimensions in martial arts athletes. We decided to explore martial arts genotypes compared to the control group and the severity of personality dimensions.

Studies have shown that there is a possibility to predict future success in sport, based on psychological factors, relatively successfully, even in an early stage of engagement in sports [42]. The latest Genome-Wide Association Study (GWAS) [43] showed a very low significant *p*-value of rs6265 *BDNF* with general risk tolerance in the general population.

Asai et al. 2020 presented the results concerning the group of 74 male judo athletes and 87 healthy male non-athletes. In the study [27], the relationship between *BDNF* gene polymorphism and sports (the main focus was on judo) was the concern. This study indicated that judo athletes, who need open skills and higher stress tolerance, presented a higher frequency of the GG genotype and a lower frequency of the AA genotype, which can strongly implicate the relationship between the *BDNF* gene polymorphism and sports. Moreover, they also emphasized that the ratio of A carriers was elevated in the group of athletes who continued sporting activity to the university level than in healthy non-athletes [44].

The research by Joffe et al. analyzed 467 non-clinical Caucasian subjects of European ethnicity who participated in the Brain Resource International Database (BRID). The study examined relationships between the *BDNF* G196A polymorphism and five-factor personality dimensions in *BDNF* A carriers. Lower total hippocampal grey matter volume was associated with higher neuroticism. These specific relationships were not present in *BDNF* Val/Val homozygotes (G196G) [45]. The study mentioned [45] showed the differences between A carriers and the GG genotype group. The first group did not have an elevated level of neuroticism in direct comparison with the second one. Some studies identified a shortage of influence of *BDNF* polymorphism and neuroticism levels in healthy volunteers [46,47]. However, some also report a small but significant elevation of neuroticism in Met carriers [47]. When considering both *BDNF* A carrier and GG groups, the studies indicate the association of higher neuroticism and higher depression trait and related symptoms of anxiety and stress, which seems to be evidence of shared genetic risk among these dimensions [48,49]. However, not enough effort has been put into investigating the relationship between genetic polymorphisms affecting psychological abilities and competitive power sports.

Our study was focused on a particular group of martial arts practitioners, and we observed that martial arts athletes’ G/G genotypes (Val66), in comparison to the control group’s G/G (Val66) genotypes, presented significantly higher scores on personality dimension “conscientiousness”. In the NEO-FFI dimension, “conscientiousness” covers personality traits as: competence, order, dutifulness, achievement striving, self-discipline, and deliberation. Our research showed that there is a possibility that the athletes who perform martial arts and present with G/G genotypes are “neurobiologically” predisposed to those achievements. An interesting question is how participation in sports and competitions influences the development of those personality features? Therefore, our future research will focus on young martial arts athletes’ G/G genotypes compared to the control group’s G/G genotypes and NEO-FFI dimensions in longitudinal research.

In comparison with the controls, the case study subjects had significantly higher scores on the extraversion/scale (M 6.89 vs. M 6.43, *p* = 0.0405) and conscientiousness/scale (M 7.23 vs. M 5.89, *p* < 0.0001). We noticed differences in the NEO-FFI scale and the analysis of interactions connected with the status of an athlete and genes. The results of 2 × 3 factorial ANOVA noticed a statistically significant effect of combined factor *BDNF* rs6265 genotype of martial arts/control (F_2,252_ = 3.11, *p* = 0.0465, η^2^ = 0.024) (Table 3, Figure 1).

Additionally, we observed that the results of 2 × 3 factorial ANOVA showed a statistically significant influence of combined factor *BDNF* rs6265 on the martial arts/control genotype (F_2,252_ = 6.16, *p* = 0.0024, η^2^ = 0.047) (Table 3, Figure 2).

Human fitness is conditioned by several environmental and genetic factors [50]. Neuronal survival, neurogenesis, growth, and synaptic plasticity are regulated by *BDNF*, whereas *BDNF* rs6265 polymorphism is connected with serum BDNF concentration that changes in response to exercise [30].

In our previous research, we also presented the influence of *BDNF* on the group of athletes. In the group of individuals practicing martial arts, the statistically significant interaction between the occurrence of T/T and A/T genotypes in *BDNF* rs10767664 was noticed, as well as increased results of the NEO FFR sten and conscientiousness scales in comparison with the control group. When comparing martial arts athletes and the control group, we also observed a statistically significant interaction between the frequency of G/G genotypes and elevated results on the NEO-FFI and conscientiousness dimension [51].

We are aware of our research’s limitations. However, as it is the only polymorphism, we observe statistical significance there.

The combination of the analysis of personality dimensions with genetics—as in the case of the polymorphism of the *BDNF* gene related to neuroplasticity—indicates that neurobiology cannot be ignored in educating sports champions. We already know that this is related to genetics. However, little is still known about the influence of personality traits on sports performance.

## 5. Conclusions

In our research, we observed that martial arts athletes’ G/G genotypes compared to the control group’s G/G genotypes presented significantly higher severity of personality dimension “conscientiousness”. This is worthy of further analysis, probably longitudinal studies on a more numerous group of athletes.

## Figures and Tables

**Table 1:** Frequency of genotypes and alleles of the *BDNF* rs6265 in a group of martial arts subjects and controls.

	Martial Arts	Controls	χ^2^ (*p*-Value)
*BDNF* rs6265			
	*n* = 106	*n* = 152	1.009 (0.6039)
G/G	76 (71.7%)	100 (65.8%)	
A/G	27 (25.5%)	47 (30.9%)	
A/A	3 (2.8%)	5 (3.3%)	
G	179 (84.4%)	247 (81.2%)	0.880 (0.3484)
A	33 (15.6%)	57 (18.8%)	
Hardy-Weinberg equilibrium			
χ^2^ (*p*-value)	0.102 (0.7498)	0.033 (0.8548)	

*p*—statistical significance, χ^2^—Chi^2^ test result, *n*—number of subjects, G/G and A/A—genotypes (homozygotes), A/G—genotype (heterozygote), G and A—alleles.

**Table 2:** Analysis of NEO Five-Factor Inventory results in martial arts subjects and controls.

NEO Five-Factor Inventory/	Martial Arts (*n* = 106) M ± SD	Control (*n* = 152) M ± SD	U Mann–Whitney Z	*p*-Value
Neuroticism/scale	4.75 ± 2.24	4.65 ± 1.92	−0.128	0.8981
Extraversion/scale	7.12 ± 1.92	6.43 ± 1.85	2.834	0.0045 *#
Openness/scale	5.01 ± 1.84	4.56 ± 1.55	2.117	0.0342 *
Agreeability/scale	6.00 ± 2.36	5.71 ± 2.06	1.038	0.2993
Conscientiousness/scale	7.26 ± 2.17	5.89 ± 1.99	5.165	0.0000 *#

M—mean, SD—standard deviation, G/G and A/A—genotypes (homozygotes), A/G—genotype (heterozygote), G and A—alleles, U Mann-Whitney Z-test. *—significant statistical differences. # Bonferroni correction was used, and the *p-*value was reduced to 0.01 (*p* = 0.05/5 (number of statistical tests conducted)).

**Table 3:** The results of 2 × 3 factorial ANOVA for martial arts subjects and controls, NEO Five-Factor Inventory scale, and *BDNF* rs6265.

NEO Five-Factor Inventory	Group	*BDNF* Gene rs6265				ANOVA		
		G/G *n* = 176 M ± SD	A/G *n* = 74 M ± SD	A/A *n* = 8 M ± SD	Factor	F (*p*-Value)	η^2^	Power (Alfa = 0.05)
Neuroticism/scale	Martial Arts (MA); *n* = 106	4.29 ± 2.02	5.85 ± 2.43	6.67 ± 2.31	intercept MA/control *BDNF* MA/control × *BDNF*	F_1,252_ = 385.18 (*p* < 0.0001) *# F_1,252_ = 2.72 (*p* = 0.1001) F_2,252_ = 6.23 (*p* = 0.0023) *# F_2,252_ = 3.11 (*p* = 0.0465) *	0.604 0.011 0.047 0.024	1.000 0.376 0.892 0.594
	Control; *n* = 152	4.55 ± 2.06	4.85 ± 1.60	4.80 ± 1.92				
Extraversion/scale	Martial Arts (MA); *n* = 106	7.39 ± 1.83	6.48 ± 2.04	6.00 ± 1.73	intercept MA/control *BDNF* MA/control × *BDNF*	F_1,252_ = 721.50 (*p* < 0.0001)*# F_1,252_ = 0.06 (*p* = 0.8039) F_2,252_ = 1.25 (*p* = 0.2866) F_2,252_ = 2.44 (*p* = 0.0894)	0.666 0.0002 0.001 0.020	1.000 0.057 0.272 0.488
	Control; *n* = 152	6.38 ± 1.92	6.53 ± 1.72	6.60 ± 1.82				
Openness/scale	Martial Arts (MA); *n* = 106	4.95 ± 1.85	5.00 ± 1.84	6.67 ± 1.15	intercept MA/control *BDNF* MA/control × *BDNF*	F_1,252_ = 501.71 (*p* < 0.0001) *# F_1,252_ = 8.62 (*p* = 0.0036) *# F_2,252_ = 0.16 (*p* = 0.8487) F_2,252_ = 2.39 (*p* = 0.0935)	0.694 0.033 0.001 0.019	1.000 0.832 0.075 0.480
	Control; *n* = 152	4.61 ± 1.56	4.55 ± 1.56	3.60 ± 1.14				
Agreeability/scale	Martial Arts (MA); *n* = 106	6.25 ± 2.37	5.55 ± 2.26	3.67 ± 1.15	intercept MA/control *BDNF* MA/control × *BDNF*	F_1,252_ = 359.03 (*p* < 0.0001) *# F_1,252_ = 0.71 (*p* = 0.3976) F_2,252_= 2.48 (*p* = 0.0857) F_2,252_ = 1.18 (*p* = 0.3091)	0.587 0.002 0.019 0.009	1.000 0.135 0.495 0.257
	Control; *n* = 152	5.81 ± 2.10	5.51 ± 1.87	5.60 ± 3.21				
Conscientiousness/scale	Martial Arts (MA); *n* = 106	7.54 ± 2.04	6.67 ± 2.20	5.67 ± 4.16	intercept MA/control *BDNF* MA/control × *BDNF*	F_1,252_ = 591.42 (*p* < 0.0001) *# F_1,252_ = 0.42 (*p* = 0.5162) F_2,252_ = 0.09 (*p* = 0.9101) F_2,252_ = 6.16 (*p* = 0.0024) *#	0.701 0.002 0.001 0.047	1.000 0.099 0.064 0.888
	Control; *n* = 152	5.57 ± 1.98	6.47 ± 1.91	6.80 ± 1.64				

*—significant result *p* = 0.05; MA—Martial Arts; M ± SD—mean ± standard deviation, G/G and A/A—genotypes (homozygotes), A/G—genotype (heterozygote), G and A—alleles, *BDNF*—Brain-Derived Neurotropic Factor, F—F ratio score, η^2^—effect size # Bonferroni correction was used, and the *p*-value was reduced to 0.01 (*p* = 0.05/5 (number of statistical tests conducted)).

**Table 4:** Post-hoc LSD (least significant difference) analysis of interactions between martial arts/-Control and *BDNF* rs6265 and neuroticism scale and conscientiousness scale.

*BDNF* rs6265 and NEO FFI Neuroticism Scale						
	{1} M = 4.29	{2} M = 5.85	{3} M = 6.67	{4} M = 4.55	{5} M = 4.85	{6} M = 4.80
Martial arts *BDNF* G/G {1}		0.0006 *#	0.0461 *	0.3963	0.1344	0.5837
Martial arts *BDNF* A/G {2}			0.5070	0.0032 *#	0.0407 *	0.2847
Martial arts *BDNF* A/A {3}				0.0742	0.1315	0.2058
Control *BDNF* G/G {4}					0.3990	0.7868
Control *BDNF* A/G {5}						0.9571
Control *BDNF* A/A {6}						
*BDNF* rs6265 and NEO FFI conscientiousness scale						
	{1} M = 7.34	{2} M = 6.67	{3} M = 5.67	{4} M = 5.67	{5} M = 6.47	{6} M = 6.80
Martial arts *BDNF* G/G{1}		0.0561	0.1183	0.0000 *#	0.0048 *#	0.4309
Martial arts *BDNF* A/G {2}			0.4190	0.0134 *	0.6858	0.8928
Martial arts *BDNF* A/A {3}				0.9353	0.5080	0.4453
Control *BDNF* G/G {4}					0.0130 *	0.1873
Control *BDNF* A/G {5}						0.7284
Control *BDNF* A/A {6}						

*—significant statistical differences, M—mean. For these variables, G/G and A/A—genotypes (homozygotes), A/G—genotype (heterozygote), G and A—alleles, *BDNF*—Brain-Derived Neurotropic Factor, # Bonferroni correction was used, and the *p*-value was reduced to 0.0083 (*p* = 0.05/6 (number of statistical tests conducted)).

## Data Availability

Not applicable.

## References

[1] Sellami M., Elrayess M.A., Puce L., Bragazzi N.L. (2022). Molecular Big Data in Sports Sciences: State-of-Art and Future Prospects of OMICS-Based Sports Sciences. Front. Mol. Biosci..

[2] Ahmetov I.I., Egorova E.S., Gabdrakhmanova L.J., Fedotovskaya O.N. (2016). Genes and Athletic Performance: An Update. Med. Sport Sci..

[3] Cervone D., Pervin L.A. (2010). Personality: Theory and Research.

[4] McCrae R.R., Costa P.T. (1997). Personality trait structure as a human universal. Am. Psychol..

[5] Piedmont R.L., Hill D.C., Blanco S. (1999). Predicting athletic performance using the five-factor model of personality. Pers. Individ. Differ..

[6] Zhang G., Chen X., Xiao L., Li Y., Li B., Yan Z., Guo L., Rost D.H. (2019). The Relationship Between Big Five and Self-Control in Boxers: A Mediating Model. Front. Psychol..

[7] Habib M.B., Waris S., Afzal S. (2019). Personality traits predict in sports performance among University athletes. Spark.

[8] Weisberg Y.J., DeYoung C.G., Hirsh J.B. (2011). Gender Differences in Personality across the Ten Aspects of the Big Five. Front. Psychol..

[9] Andersen B.P. (2020). Ethnic group differences in the general factor of personality (GFP) are opposite to that which would be predicted by differential-K theory. Pers. Individ. Differ..

[10] Singh K. (2017). Comparative study on personality amongst athletes of individual and team sports. Int. J. Dev. Res..

[11] Khan B., Ahmed A., Abid G. (2016). Using the ‘Big-Five’-For Assessing Personality Traits of the Champions: An Insinuation for the Sports Industry. Pak. J. Commer. Soc. Sci..

[12] Silverman M.N., Deuster P.A. (2014). Biological mechanisms underlying the role of physical fitness in health and resilience. Interface Focus.

[13] Kitahara M., Inoue T., Mani H., Takamatsu Y., Ikegami R., Tohyama H., Maejima H. (2021). Exercise and pharmacological inhibition of histone deacetylase improves cognitive function accompanied by an increase of gene expressions crucial for neuronal plasticity in the hippocampus. Neurosci. Lett..

[14] Chou C.-C., Chien L.-Y., Lin M.-F., Wang C.-J., Liu P.-Y. (2022). Effects of Aerobic Walking on Memory, Subjective Cognitive Complaints, and Brain-Derived Neurotrophic Factor Among Older Hypertensive Women. Biol. Res. Nurs..

[15] Simão A.P., Mendonça V.A., Avelar N.C.P., Da Fonseca S.F., Santos J.M., De Oliveira A.C.C., Tossige-Gomes R., Ribeiro V.G.C., Neves C.D.C., Balthazar C.H. (2019). Whole Body Vibration Training on Muscle Strength and Brain-Derived Neurotrophic Factor Levels in Elderly Woman With Knee Osteoarthritis: A Randomized Clinical Trial Study. Front. Physiol..

[16] de Las Heras B., Rodrigues L., Cristini J., Weiss M., Prats-Puig A., Roig M. (2022). Does the Brain-Derived Neurotrophic Factor Val66Met Polymorphism Modulate the Effects of Physical Activity and Exercise on Cognition?. Neuroscientist.

[17] Müller P., Duderstadt Y., Lessmann V., Müller N.G. (2020). Lactate and BDNF: Key Mediators of Exercise Induced Neuroplasticity?. J. Clin. Med..

[18] Tsatsoulis A., Fountoulakis S. (2006). The Protective Role of Exercise on Stress System Dysregulation and Comorbidities. Ann. N. Y. Acad. Sci..

[19] Ryan S.M., Nolan Y.M. (2016). Neuroinflammation negatively affects adult hippocampal neurogenesis and cognition: Can exercise compensate?. Neurosci. Biobehav. Rev..

[20] Packer N., Pervaiz N., Hoffman-Goetz L. (2010). Does exercise protect from cognitive decline by altering brain cytokine and apoptotic protein levels? A systematic review of the literature. Exerc. Immunol. Rev..

[21] Caldeira M.V., Melo C.V., Pereira D.B., Carvalho R.F., Carvalho A.L., Duarte C.B. (2007). BDNF regulates the expression and traffic of NMDA receptors in cultured hippocampal neurons. Mol. Cell. Neurosci..

[22] Neeper S.A., Góauctemez-Pinilla F., Choi J., Cotman C. (1995). Exercise and brain neurotrophins. Nature.

[23] Rasmussen P., Brassard P., Adser H., Pedersen M.V., Leick L., Hart E., Secher N.H., Pedersen B.K., Pilegaard H. (2009). Evidence for a release of brain-derived neurotrophic factor from the brain during exercise. Exp. Physiol..

[24] Dinoff A., Herrmann N., Swardfager W., Liu C.S., Sherman C., Chan S., Lanctôt K.L. (2016). The Effect of Exercise Training on Resting Concentrations of Peripheral Brain-Derived Neurotrophic Factor (BDNF): A Meta-Analysis. PLoS ONE.

[25] Dinoff A., Herrmann N., Swardfager W., Lanctôt K.L. (2017). The effect of acute exercise on blood concentrations of brain-derived neurotrophic factor in healthy adults: A meta-analysis. Eur. J. Neurosci..

[26] Gomes de Assis G., Cięszczyk P. (2020). Exercise—A Unique Endogenous Regulator of Irisin, BDNF, Leptin and Cortisol against Depression. Balt. J. Health Phys. Act..

[27] Asai T., Abe D., Doi H., Tanaka C., Ohishi K., Maeda H., Wada T., Takahashi Y., Nakahata Y., Shinohara K. (2020). Characteristics of the BDNF Val66Met Polymorphism in Competitive Swimmers and Judo Athletes. Acta Med. Nagasaki..

[28] Joffe R.T., Gatt J.M., Kemp A.H., Grieve S., Dobson-Stone C., Kuan S.A., Schofield P.R., Gordon E., Williams L.M. (2009). Brain derived neurotrophic factor Val66Met polymorphism, the five factor model of personality and hippocampal volume: Implications for depressive illness. Hum. Brain Mapp..

[29] Willis-Owen S.A., Fullerton J., Surtees P.G., Wainwright N.W., Miller S., Flint J. (2005). The Val66Met Coding Variant of the Brain-Derived Neurotrophic Factor (BDNF) Gene Does Not Contribute Toward Variation in the Personality Trait Neuroticism. Biol. Psychiatry.

[30] Lang U.E., Hellweg R., Kalus P., Bajbouj M., Lenzen K.P., Sander T., Kunz D., Gallinat J. (2005). Association of a functional BDNF polymorphism and anxiety-related personality traits. Psychopharmacology.

[31] Sen S., Nesse R., Stoltenberg S.F., Li S., Gleiberman L., Chakravarti A., Weder A.B., Burmeister M. (2003). A BDNF Coding Variant is Associated with the NEO Personality Inventory Domain Neuroticism, a Risk Factor for Depression. Neuropsychopharmacology.

[32] Kendler K.S., Neale M.C., Kessler R.C., Heath A.C., Eaves L.J. (1994). The Clinical Characteristics of Major Depression as Indices of the Familial Risk to Illness. Br. J. Psychiatry.

[33] Anastasia A., Deinhardt K., Chao M., Will N.E., Irmady K., Lee F.S., Hempstead B.L., Bracken C. (2013). Val66Met polymorphism of BDNF alters prodomain structure to induce neuronal growth cone retraction. Nat. Commun..

[34] Arumuggam N., Bhowmick N.A., Rupasinghe H.P.V. (2015). A Review: Phytochemicals Targeting JAK/STAT Signaling and IDO Expression in Cancer. Phytotherapy Res..

[35] Ferris L.T., Williams J.S., Shen C.-L. (2007). The Effect of Acute Exercise on Serum Brain-Derived Neurotrophic Factor Levels and Cognitive Function. Med. Sci. Sports Exerc..

[36] Egan M.F., Kojima M., Callicott J.H., Goldberg T.E., Kolachana B.S., Bertolino A., Zaitsev E., Gold B., Goldman D., Dean M. (2003). The BDNF val66met Polymorphism Affects Activity-Dependent Secretion of BDNF and Human Memory and Hippocampal Function. Cell.

[37] Haslacher H., Michlmayr M., Batmyagmar D., Perkmann T., Ponocny-Seliger E., Scheichenberger V., Pilger A., Dal-Bianco P., Lehrner J., Pezawas L. (2015). Physical Exercise Counteracts Genetic Susceptibility to Depression. Neuropsychobiology.

[38] Bath K.G., Lee F.S. (2006). Variant BDNF (Val66Met) impact on brain structure and function. Cogn. Affect. Behav. Neurosci..

[39] Zoladz J.A., Pilc A., Majerczak J., Grandys M., Zapart-Bukowska J., Duda K. (2008). Endurance training increases plasma brain-derived neurotrophic factor concentration in young healthy men. J. Physiol. Pharmacol..

[40] Pedersen B.K., Pedersen M., Krabbe K.S., Bruunsgaard H., Matthews V.B., Febbraio M.A. (2009). Role of exercise-induced brain-derived neurotrophic factor production in the regulation of energy homeostasis in mammals. Exp. Physiol..

[41] Knaepen K., Goekint M., Heyman E.M., Meeusen R. (2010). Neuroplasticity—Exercise-induced response of peripheral brain-derived neurotrophic factor: A systematic review of experimental studies in human subjects. Sports Med..

[42] Yarrow J.F., White L.J., McCoy S.C., Borst S.E. (2010). Training augments resistance exercise induced elevation of circulating brain derived neurotrophic factor (BDNF). Neurosci. Lett..

[43] Walsh J.J., Scribbans T.D., Bentley R.F., Kellawan J.M., Gurd B., Tschakovsky M.E. (2016). Neurotrophic growth factor responses to lower body resistance training in older adults. Appl. Physiol. Nutr. Metab..

[44] Walsh J.J., Tschakovsky M.E. (2018). Exercise and circulating BDNF: Mechanisms of release and implications for the design of exercise interventions. Appl. Physiol. Nutr. Metab..

[45] Donati F., Sian V., Biasini G.M., de la Torre X., Folchitto F., Botrè F. (2021). Serum Levels of Brain-Derived Neurotrophic Factor and Other Neurotrophins in Elite Athletes: Potential Markers of the Use of Transcranial Direct Current Stimulation in Sport. Front. Sports Act. Living.

[46] Ribeiro D., Petrigna L., Pereira F.C., Muscella A., Bianco A., Tavares P. (2021). The Impact of Physical Exercise on the Circulating Levels of BDNF and NT 4/5: A Review. Int. J. Mol. Sci..

[47] Costa P.T., McCrae R.R. (2008). The SAGE Handbook of Personality Theory and Assessment: Volume 2—Personality Measurement and Testing.

[48] Yperen N.W.V. (2009). Why some make it and others do not: Identifying psychological factors that predict career success in professional adult soccer. Sport Psychol..

[49] Linnér R.K., Biroli P., Kong E., Meddens S.F.W., Wedow R., Fontana M.A., Lebreton M., Tino S.P., Abdellaoui A., Hammerschlag A.R. (2019). Genome-wide association analyses of risk tolerance and risky behaviors in over 1 million individuals identify hundreds of loci and shared genetic influences. Nat. Genet..

[50] De Moor M.H.M., Spector T.D., Cherkas L.F., Falchi M., Hottenga J.J., Boomsma D.I., de Geus E. (2007). Genome-Wide Linkage Scan for Athlete Status in 700 British Female DZ Twin Pairs. Twin Res. Hum. Genet..

[51] Niewczas M., Król P., Czarny W., Bajorek W., Rzepko M., Drozd S., Płonka A., Drozd M., Czaja R., Błach W. (2021). Association Analysis of Polymorphic Variants of the *BDNF* Gene in Athletes. Genes.

